# Comparison of digital image analysis and visual scoring of KI-67 in prostate cancer prognosis after prostatectomy

**DOI:** 10.1186/s13000-015-0294-0

**Published:** 2015-06-13

**Authors:** Patrice Desmeules, Hélène Hovington, Molière Nguilé-Makao, Caroline Léger, André Caron, Louis Lacombe, Yves Fradet, Bernard Têtu, Vincent Fradet

**Affiliations:** Department of Surgery/Urology, Faculty of Medicine, Laval University, Québec, Canada; Department of Medicine, Faculty of Medicine, University of Montreal, Montreal, Canada; Cancer Research Centre, CHU de Québec, Québec, Canada; Population Health Unit (URESP), Centre de recherche FRQS du Centre hospitalier affilié universitaire de Québec, Québec, Canada; Anatomic Pathology and Cytology Department, Hôpital du St-Sacrement, Centre Hospitalier Universitaire (CHU) de Québec, Laval University, Québec, Canada; Centre de recherche en cancérologie de l’Université Laval, Centre Hospitalier Universitaire de Québec – pavillon L’Hôtel-Dieu de Québec, 10 rue McMahon, Québec, QC G1R3S1 Canada

**Keywords:** Prostate cancer, Proliferation, Biochemical recurrence, Mortality, Digital image analysis

## Abstract

**Background:**

The tumor proliferative index marker Ki-67 was shown to be associated with clinically significant outcomes in prostate cancer, but its clinical application has limitations due to lack of uniformity and consistency in quantification. Our objective was to compare the measurements obtained with digital image analysis (DIA) versus virtual microscopy (visual scoring (VS)).

**Methods:**

To do so, we compared the measurement distributions of each technique and their ability to predict clinically useful endpoints. A tissue microarray series from a cohort of 225 men who underwent radical prostatectomy was immunostained for Ki-67. The percentage of Ki-67 positive nuclei in malignant cells was assessed both by VS and DIA, and a H–score was calculated. The distribution and predictive ability of these scoring methods to predict biochemical recurrence (BCR) and death from prostate cancer (DPCa) were compared using Mann–Whitney test and C-index.

**Results:**

The measurements obtained with VS were similar to the DIA measurements (p = 0.73) but dissimilar to the H-score (p < 0.001). Cox regression models showed that Ki-67 was associated with BCR (HR 1.46, 95 % CI 1.10-1.94) and DPCa (HR 1.26, 95 % CI 1.06-1.50). C-indexes revealed that Ki-67 was a better predictor of DPCa (0.803, 0.8059 and 0.789; VS, DIA and H-score, respectively) than of BCR (0.625, 0.632 and 0.604; VS, DIA and H-score, respectively).

**Conclusion:**

The measurement distributions and the predictive abilities of VS and DIA were similar and presented the same predictive behaviour in our cohort, supporting the role of Ki-67 proliferative index as an important prognostic factor of BCR and DPCa in prostate cancer post RP.

**Virtual Slides:**

The virtual slide(s) for this article can be found here: http://www.diagnosticpathology.diagnomx.eu/vs/6656878501536663

**Electronic supplementary material:**

The online version of this article (doi:10.1186/s13000-015-0294-0) contains supplementary material, which is available to authorized users.

## Background

Prostate cancer (PCa) is a major health problem, being the most frequent cancer in men and a leading cause of cancer-related death in North America [1]. Despite an increase in the ability to detect cancer, the clinical behavior of prostate cancer remains hard to predict and ranges from indolent course to aggressive evolution with metastasis and death. Gleason score, tumor stage and pre-operative PSA are the only current prognostic factors available, and are used, alone or in combination, to predict the evolution of this disease [2, 3]. Nonetheless, none of these factors has enough prognostic ability to identify which patients should be additionally treated, given their individual risk of biochemical recurrence or death after radical prostatectomy.

In order to avoid over- or under-treatment, the ability of several histologic measurements (including histological type, androgen receptor status [4], expression of specific cancer markers such as p53, Ras oncogene, and BCL2 [5–7]) to improve the prediction of individual prognosis have been explored. The expression of the Ki-67 antigen, a nuclear protein expressed during the G1, S, G2 and M phases of the cell cycle but not during the resting phase (G0) [8, 9], has emerged as the preferred marker of tumor proliferation in different tissue types [10–13], offering interesting predictive value in breast cancer [14] and neuroendocrine digestive tumors [15]. Clinical studies have also shown the ability of this marker to distinguish aggressive from non-aggressive PCa in cohorts with different treatment modalities [16–26]. However, the integration of this analysis in the current predictive arsenal to determine the appropriate treatment strategy to adopt given the risk of progression remains a challenge, mainly due to issues related to the reproducibility of measurement methods and application of a threshold value.

Manual counting of a tumor proliferation index is technically long and tedious and also subject to intra- and inter-observer variability. In this context, digital image analysis (DIA) offers a potential adjunct to conventional visual scoring with the promise to increase reproducibility, accuracy and rapidity of the quantification process. Indeed, increasing reports of DIA reproducing visual scoring at an acceptable level are found [27, 28]. However, it is unknown whether one method is superior to the other to assess Ki-67 index, even in tumors with a larger hindsight on DIA use such as breast cancer [29]. Such method still needs to be validated against traditional visual assessment for a broader range of patients and tissues.

Our objective was to compare the Ki-67 measurements obtained with the DIA and the visual scoring methods. To achieve this objective, we first compared the measurement distributions of the two scoring methods and then compared their ability to predict clinically useful endpoints (biochemical failure and death by prostate cancer) over time. We took advantage of a well-constructed and characterized cohort of PCa patients who underwent radical prostatectomy and benefited from extended follow-up.

## Methods

### Patients and data collection

Patients included in this study were selected randomly from a cohort of Caucasian men who were treated by radical prostatectomy (RP) for localized prostate cancer at L’Hôtel-Dieu de Québec (Québec city, Canada) between 1990 and 2002. Men were included if they had a prostate adenocarcinoma of any stage treated with RP. Patients who received neo-adjuvant androgen deprivation therapy were excluded. All men provided informed written consent to participate in this study. The Institutional Ethical Research Committee approved the protocol.

The patient’s medical files were reviewed to collect clinical characteristics (age, stage, histology, Gleason score, preoperative and follow-up serum PSA levels, status at last follow-up). Clinical follow-up data were regularly updated until June 2012 to document the occurrence of progression or death after surgery. Clinical follow-up was performed at least every year and included PSA testing and physical examination. PSA recurrence was defined as two consecutive PSA values of at least 0.3 ng/mL, or one PSA value of at least 0.3 ng/mL followed by androgen deprivation therapy or radiation therapy, or PSA value less than 0.3 ng/mL but with an androgen-deprivation therapy. Biochemical recurrence (BCR) after radical prostatectomy was defined as the period of time elapsed between the prostatectomy and the first date of meeting the criteria of PSA recurrence. Prostate cancer-specific mortality was defined as the interval of time between the prostatectomy and death from prostate cancer. For the purpose of statistical analysis, Gleason scores were dichotomized (<7 vs ≥ 7) to group patients with intermediate and less favorable prognostic. Pathological stages were also grouped (pT2 vs pT3) to compare between organ-confined versus extra-prostatic disease.

### TMA construction

For the preparation of tissue microarrays (TMA), one to three paraffin blocks from each tumor was selected. Six representative 0,6 mm tumor cores were taken and placed 0,4 mm apart on a recipient paraffin block along with appropriate alignment and staining controls using a tissue arrayer (Beecher Instruments, Sun Prairie, WI, USA). All primary tumor slices were examined and graded by a pathologist (BT). Spots were selected from haematoxylin and eosin slides to represent the proportion of each Gleason patterns amongst dominant nodules and include a tertiary pattern when present. Gleason scores were also evaluated on TMA cores to ensure the representativeness of the sample with regard to the prostatectomy specimen.

### Immunohistochemistry and slide digitization

Sections five micron-thick were cut from the tissue array blocks to perform immunohistochemistry. Sections were deparafinized and heat-induced antigen retrieval was performed by microwave pre-treatment in citrate (0.01 M, pH 6.0). Endogenous peroxidase was blocked by pre-incubation with 3 % hydrogen peroxide in phosphate-buffered saline for 10 minutes. Sections were then incubated with a monoclonal mouse anti-human Ki-67 antibody (Clone MIB-1; Dako, Carpinteria, CA, USA) at room temperature for 1 h at a 1/400 dilution. Chromogenic detection was carried out using a peroxidase-conjugated secondary antibody and DAB reagents provided with the IDetect Super Stain System HRP (ID Labs, London, ON, Canada), and slides were counter-stained with Harris’ hematoxylin. Positive controls were slides of lympho-epithelial tissue. Phosphate buffered saline was used instead of primary antibody in negative controls.

Digital images of IHC-stained TMA slides were obtained at 20× magnification using a slide scanner (NanoZoomer 2.0-HT; Hamamatsu, Bridgewater, NJ, USA) and visualized with the software ndpi.viewer (ver1.2.25; Olympus). These images were used both for the visual scoring and the digital image analysis.

### Visual scoring

Ki-67 nuclear positivity was evaluated on digital images by two independent observers (PD, HH) blinded to the clinical data. The total number of Ki-67 positive tumor nuclei was counted in malignant cells on each individual TMA spot, regardless of the intensity of immunostaining or the Gleason score. This count was reported on the total number of malignant cells, calculated by using a 100-cell template moved over the whole tissue core. A TMA spot was rejected when neoplastic glands covered less than 30 % of the tissue. Moreover, a minimum number of 500 tumor cells per patient was require to keep the sample for analysis. The mean percentage of Ki-67 positive cells for each patient was calculated and used in further analysis as the Ki-67 labeling index.

### Digital image analysis

Automated-IHC measurements were performed using Calopix software (developed by TRIBVN [Chatillon, France] and distributed by Agfa Healthcare [Toronto, ON, Canada]). For the purpose of tissue recognition and segmentation, the Ilastik 5.0 Interactive Learning and Segmentation Toolkit within Calopix was used to create a tissue mask and conserve only the malignant epithelial component for analysis (see Additional file 1: Figure S1). Then, the Calopix «immuno-object» software was applied to each segmentation result. The algorithm used allows recognition of individual nuclei («objects») by isolating brown (DAB stained) and blue (hematoxilin counter-stained) nuclei, and report their numbers. Within the algorithm, immunostaining is divided in intensity categories (0 = none, 1 = weak, 2 = moderate, 3 = strong), for which thresholds are set on visual appreciation. Segmentation and quantification algorithms were performed at 10× resolution, and all the results were reviewed visually. When the tissue segmentation was judged unsatisfactory (poor isolation of tumor glands), a gross manual segmentation was done before re-launching both Ilastik and immuno-object algorithms successively. For each tissue core, the total number of objects detected (i.e. tumor cell nuclei), the percent of immunostained objects in each of the four staining intensity categories and the global percentage of immunostained objects were computed. Those data were used to calculate a mean H-score for each patient [30]. This compounded score is obtained when the percentage of immunostained objects in each intensity class is multiplied by the class’ category value (0, 1, 2 or 3). The results are then added, with a maximum value of 300.

### Statistical analysis

First, Mann–Whitney-Wilcoxon tests were used to compare the distribution of the mean number of Ki-67 positive cells per patient obtained with the visual and automated methods. Second, Cox regression models were used to estimate the effect of the Ki-67 labeling index on the BCR or DPCa after RP according to the measurements obtained with the two methods. PSA, Gleason score and age were used as the adjustment covariates in multivariable analyses. In addition, we created a high-risk predictor by identifying patients who had at least one high-risk feature: high pathological stage (pT3), nodal involvement and presence of positive margins (pT3N + M+). Third, we built ROC curves, assessed the AUC at the 12.5^th^ year after the RP and estimated the C-index over 12.5 years of follow-up after the RP. The C-index is used to assess the prediction accuracy of three measures of Ki-67 over time on the occurrence of BCR and DPCa. The choice of this date (12.5 years) is justified by the fact that after this time, the prediction accuracy is confounded by naturally occurring death from other causes. The statistical analyses were conducted using R-software (Version R.3.0.2; Vienna, Austria) using a two-sided alpha value of 0.05 to declare statistical significance.

## Results

### Population and clinicopathological characteristics

Among the 251 eligible patients, the Ki-67 labeling index could be evaluated in 225 patients by visual and digital assessment. The remaining 26 patients were either missing the visual or digital assessment because of inadequate staining or technical issues. Patients’ characteristics are detailed in Table 1. Stage pT3 disease was noted in 68 % of patients and Gleason score ≥ 7 in 59.1 %. The mean age at diagnosis was 63.2 years (SD: 6.10). The mean follow-up duration until BCR and DPCa was 6.8 years (SD: 4.50) and 10.6 years (SD: 3.06), respectively. During this time, 106 (47.1 %) patients experienced BCR, 44 (19.5 %) patients died, of which 10 (4.4 %) died of prostate cancer.

**Table 1:** Baseline characteristics of study subjects

Characteristics	Patients (n = 225)
Age (years), mean (SD)	63.2 (6.1)
Pre-op PSA (ng/mL), mean (SD)	12.8 (15.5)
Clinical tumor stage, n (%)	
T1	73 (32.5)
T2	142 (63.1)
T3	5 (2.2)
NA	5 (2.2)
Gleason score, n (%)	
≤6	92 (40.9)
7	93 (41.3)
≥8	40 (17.8)
Pathological stage, n (%)	
pT2a	7 (3.1)
pT2b	3 (1.3)
pT2c	60 (26.7)
pT3a	100 (44.4)
pT3b	55 (24.4)
Margin status, n (%)	
0 (negative)	71 (31.6)
1 (positive)	154 (68.4)
Nodal status, n (%)	
N0	186 (82.7)
N1	39 (17.3)
High risk score (pT3N + M+), n (%)	
0	32 (14.2)
1	192 (85.8)
NA	1 (0.0)
Biochemical recurrence, n (%)	
No	119 (52.9)
Yes	106 (47.1)
Death by prostate cancer, n (%)	
No	215 (95.6))
Yes	10 (4.4)
Death by other causes, n (%)	
No	191 (84.9))
Yes	34 (15.1)
Death – all causes, n (%)	
No	181 (80.4)
Yes	44 (19.6)
Time to BCR (years), mean (SD)	6.8 (4.5)
Time to DPCa (years), mean (SD)	10.6 (3.1)

DIA: digital image analysis; PSA: prostate-specific antigen; BCR: biochemical recurrence; DPCa: Death by prostate cancer; SD: standard deviation; NA: non available; N+: nodal involvement; M+: positive margins

### Immunohistochemistry

Ki-67 exhibited the expected nuclear staining and was globally expressed at a low level throughout tumor cells, and with considerable tissue heterogeneity. Using visual scoring as the gold standard, the mean percentage of Ki-67 nuclear labeling was 2.23 (SD: 1.98) with a median of 1.61 (IQR: 0.71-3.23). With DIA, the mean Ki-67 percentage was 2.05 (SD: 1.74) with a median of 1.48 (IQR: 0.86-2.83). Additional file 1: Figure S1 shows an example of the tissue mask and the result of the analysis performed with DIA. Overall, due to tissue heterogeneity and complexity, poor discrimination of tumor glands from high-grade PIN and in some case, the stromal component, manual correction of segmentation and/or analysis algorithm was performed on 38.1 % of the tissue cores.

### Comparison between visual assessment and DIA

**Fig. 1:**
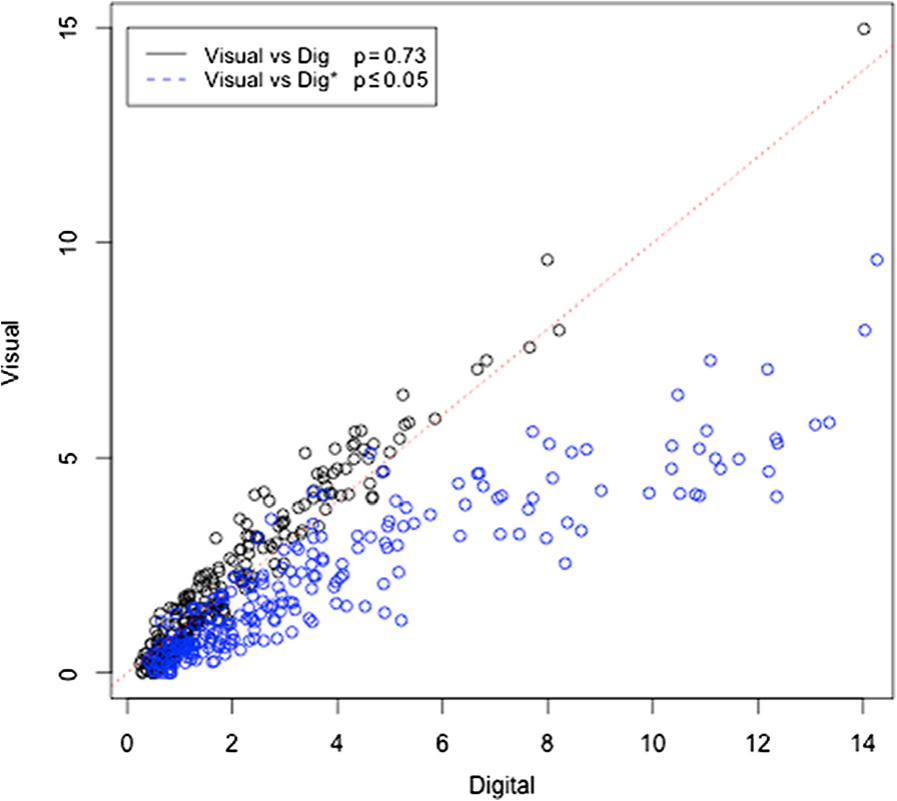
Comparison of measurements methods. Ki-67 measures obtained with digital image analysis (DIA) versus visual scoring (black dots), and weighted DIA (H-score) versus visual scoring (blue dots) were compared using a Mann–Whitney-Wilcoxon test. Visual: visual scoring, Dig: DIA and Dig*: DIA compounded measures of Ki-67 labeling index according to staining intensity (H-score)

A comparison of measurements was made between the visual assessment of Ki-67 and the DIA. In Fig. 1, the black dots represent the comparison between the visual measures versus the global DIA measures, showing no significant difference between the two methods (p = 0.73). In contrast, a significant difference was observed between the weighted DIA measurements (H-Score) and the visual assessments (p ≤ 0.001; blue dots).

### Association between Ki-67 labeling index and biochemical recurrence

As most studies published on the association between Ki-67 and BCR used a binary model for Ki-67, we determined if this association was reproducible in our cohort. In Additional file 5: Table S1, we first verified the supporting hypothesis of the Cox model. To do so, we demonstrated that the covariates used in our model (continuous or binary Ki-67 labeling index, PSA score, Gleason score, nodal involvement and presence of margin involvement in high stage tumors [high-risk score: pT3N + M+] and age) all verified the proportionality assumption. In Additional file 2: Figure S2, we demonstrate that age, Gleason score and PSA are log-linear. Cox model with a binary Ki-67 (cutoff = 1.7 %) showed that both the non-adjusted and adjusted models were significantly associated with BCR (HR 1.7, 95 % CI 1.53–2.52 and HR 1.87, 95 % CI 1.25 – 2.81, respectively), thus successfully reproducing previously published observations.

We then set up to determine if Ki-67 used as a continuous variable could also be predictive of BCR. For this procedure, we verified if Ki-67 staining was log-linear. As shown in Additional file 3: Figure S3, log-linearity for Ki-67 was only obtained with a square-root transformation. Table 2 shows that the association between Ki-67 and BCR is not significant in the univariate Cox model (HR 1.29, 95 % CI 0.97 – 1.73), but becomes significant after incorporating the adjustment covariates (HR 1.46, 95 % CI 1.10-1.94). Furthermore, we noted that the variance of the estimates of the continuous model of Ki-67 is smaller than the binary model, both for the univariate (var = 0.147 versus 0.199, respectively) and multivariate (var = 0.145 versus 0.206, respectively) analysis.

**Table 2:** Ki-67 as a predictor of clinically significant outcomes

Outcome: Biochemical recurrence			
	HR	95 % CI	p-value
Univariate model			
Ki-67*^,&^	1.29	0.97-1.73	0.019
Multivariate model			
Ki-67*^&^	1.46	1.10-1.94	0.009
PSA (μg/L)	1.02	1.01-1.03	<0.001
Gleason Score^&^	1.09	0.94-1.26	0.25
pT3N + M+	1.47	1.17-1.84	<0.001
Age (years)	0.96	0.93-0.99	0.02
Outcome: Death from prostate cancer			
	HR	95 % CI	p-value
Univariate model			
Ki-67^&^	1.19	1.01-1.41	0.040
Multivariate model			
Ki-67^&^	1.26	1.06-150	0.010
PSA (μg/L)	1.01	0.98-1.04	0.577
Gleason Score^&^	1.30	0.79-2.14	0.293
pT3N + M+	3.49	1.19-10.23	0.022
Age (years)	0.97	0.86-1.08	0.554

Ki-67*: The square root of Ki-67; PSA: Prostate-specific antigen; HR: Hazard ratio; CI: Confidence interval

^&^Ki-67 was assessed as a continuous variable. Gleason score was dichotomized to <7 vs ≥ 7

**Fig. 2:**
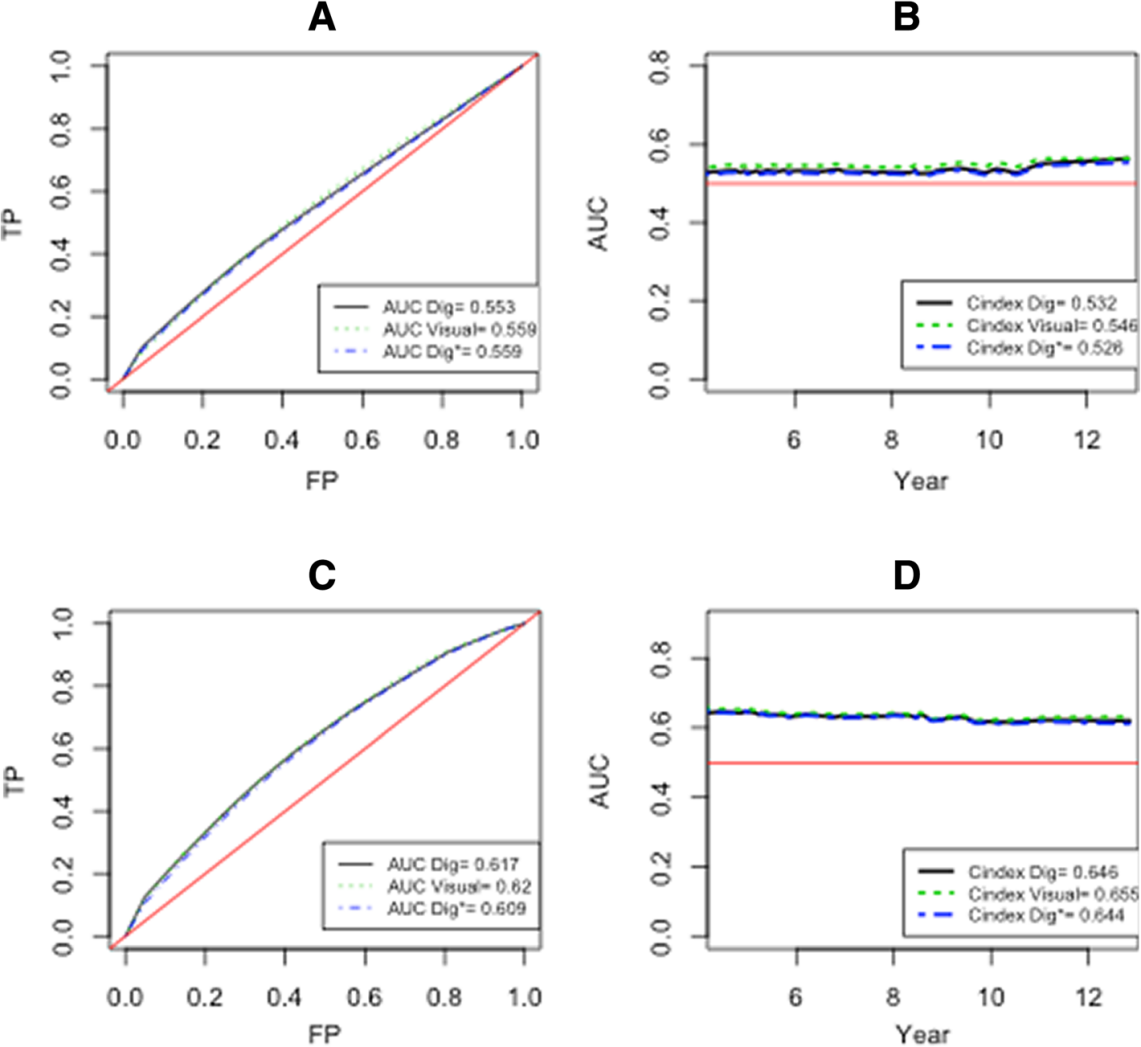
Prediction accuracy of Cox regression model (outcome: biochemical recurrence). The graphs (**a**) and (**c**) represent the ROC curve at the 12.5^th^ year after radical prostatectomy for the non-adjusted and adjusted model, respectively. The graphs (**b**) and (**d**) depict the C-index for the non-adjusted and adjusted model, respectively assessed on 12.5 years after radial prostatectomy. The red curve represents the chance while the black, blue and green line represents the measures obtained with DIA, H-score and the visual scoring, respectively. TP = true positive; FP = false positive

Nonetheless, Fig. 2 shows that Ki-67 non-adjusted (A) or adjusted (C), used as a continuous variable is not a good predictor of BCR at the 12.5^th^ year after RP. The conclusion holds even when we evaluate its ability to predict BCR over 12.5 years. Indeed the C-indexes of the adjusted model (D) of the visual scoring, DIA and weighted DIA (H-score) computed over 12.5 years are, respectively, 0.549, 0.650 and 0.640, these prediction probabilities not being significantly different from chance alone.

### Association between Ki-67 labeling index and death by prostate cancer

**Fig. 3:**
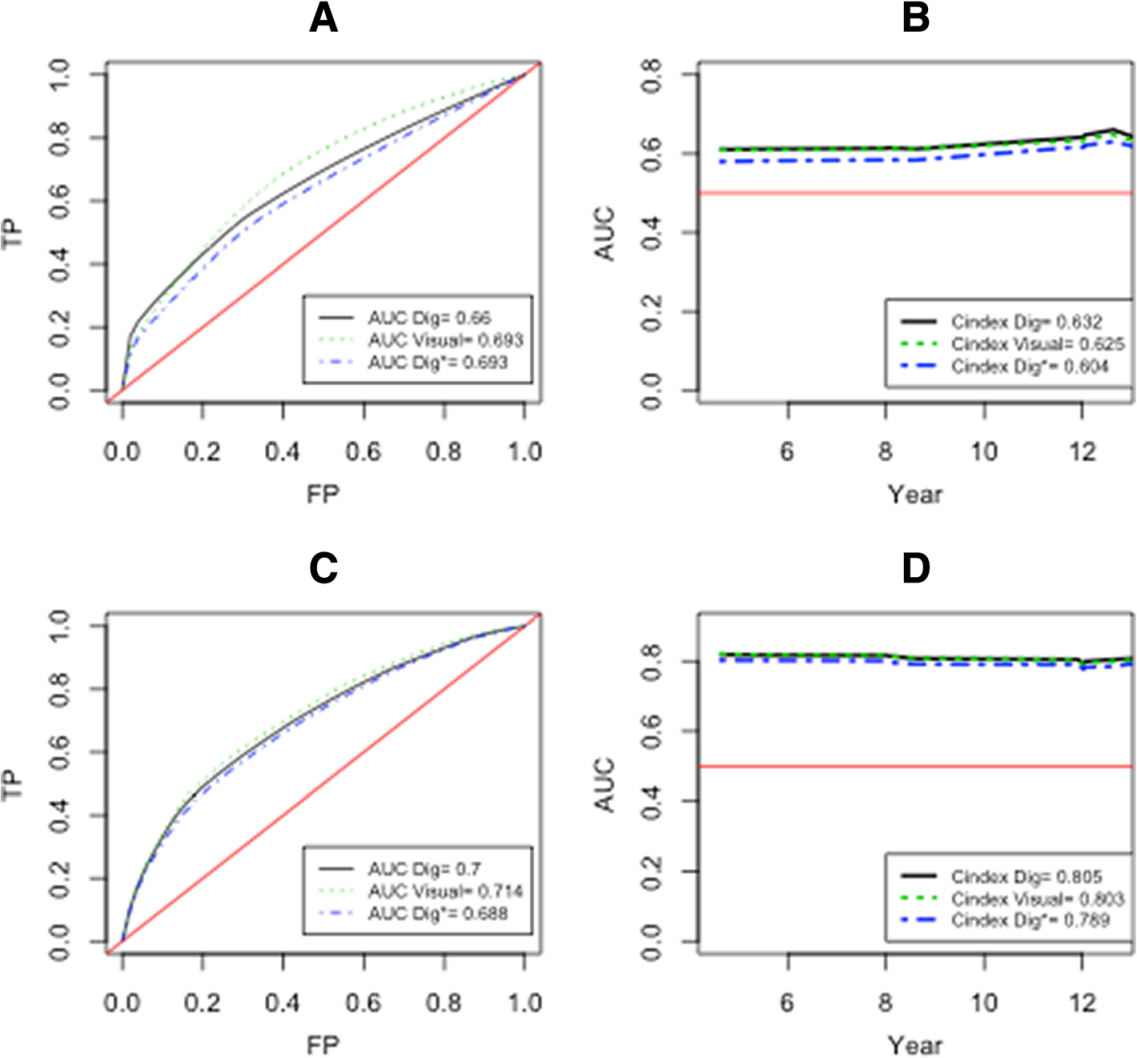
Prediction accuracy of Cox regression model (outcome: death by prostate cancer). The graphs (**a**) and (**c**) represent the ROC curve at the 12.5^th^ year after radical prostatectomy for the non-adjusted and adjusted model, respectively. The graphs (**b**) and (**d**) depict the C-index for non-adjusted and adjusted models, respectively assessed on 12.5 years after radial prostatectomy. The red curve represents the chance, while the black, blue and green line represents the measures obtained with DIA, H-score and the visual scoring, respectively. TP = true positive; FP = false positive

In our second type of models, we considered the association between Ki-67 and death by prostate cancer. Since the continuous Ki-67 modeling was superior to the binary model, we performed the following tests only with the continuous modeling. In this model, we also used Ki-67, PSA score, Gleason score, high-risk score (pT3N + M+) and age as adjustment covariates. For this model, all those covariates verified the proportionality (Additional file 5: Table S1) and log-linearity assumptions (Additional file 4: Figure S4). The association between Ki-67 and DPCa was positive in the univariate (HR 1.19, 95 % CI 1.01 – 1.41) and multivariate (HR 1.26, 95 % CI 1.06-1.50) models (Table 3). The ability of the adjusted Ki-67 is better than the non-adjusted Ki-67 to predict DPCa at the end of the follow-up period (i.e. 12.5 years) (Fig. 3C vs Fig. 3A). Furthermore, we also observed that the ability of the non-adjusted measure of Ki-67 to predict DPCa over time (Fig. 3B) is weak, as the C-indexes computed over 12.5 years with the measures obtained by visual scoring, DIA and weighted DIA, are only 0.632, 0.625 and 0.604, respectively. On the other hand, the ability of the adjusted Ki-67 to predict DPCa is good (Fig. 3D), with C-indexes (computed over 12.5 years) for the measurements done by visual scoring, DIA and weighted DIA of 0.803, 0.805 and 0.789, respectively. The mean values of C-index obtained by the bootstrap method are depicted in Table 3. We noted that the three measurements in adjusted models are good predictors of the DPCa; however, only the measurement distributions of the VS and DIA are significantly similar (Fig. 1) and they have the same C-index value.

**Table 3:** Confidence intervals of the C-indices for the multivariate Cox regression models predicting death by prostate cancer*

	Mean	95 % CI
C-index VS	0.850	0.728-0.959
C-index DIA	0.850	0.734-0.960
C-index H-score	0.834	0.724-0.950

CI: Confidence interval; VS: visual scoring

DIA: digital image analysis

*The CI were constructed using the bootstrap method

(300 iterations)

## Discussion

Uncontrolled proliferation is a hallmark of malignancy and the measurement of Ki-67 antigen by immunohistochemistry is the most widely performed assessment of the proliferative potential of tumors. Several studies are supporting a role for this marker in the prediction of clinically significant outcomes in prostate cancer, such as biochemical recurrence and death, but it is not routinely used in PCa prognostic models. As with other histopathological factors, clinical adoption is challenged by the weak reproducibility in measurement methods, poor standardization of assays and the need for clinical agreement on an appropriate measurement method, as noted by a group evaluating Ki-67 measurement in breast cancer [29]. Variability between pathologists’ assessments of Ki-67 index was reported to be high, reflecting the differences inherent to subjective counting in terms of positivity threshold interpretation, field selection as well as other factors [31]. In the absence of harmonized methodology, the integration of Ki-67 in a prognostic composite index is very difficult.

In this TMA study of PCa patients who underwent RP, we measured the expression level of Ki-67 in 225 patients by both DIA and visual scoring. With the DIA method we used, the mean labeling index (2.05 %) was similar to the one measured by visual scoring (2.23 %). Nonetheless, since similarities in the labeling index measurements between the two methods does not guarantee similar associations with clinical outcomes, we compared them based on time-dependent AUC (C-index) and show that both methods performed similarly to predict BCR and DPCa. Not only was the DIA able to reproduce the relationship with clinico-pathological outcomes obtained by visual scoring of Ki-67 index, it also had a similar pattern of prediction. Indeed, the C-index indicates that both methods allow one to discriminate between those who will die from their prostate cancer from those who won’t, even when we take into account the heterogenetity of the population, with an error rate as low as 19 %. Therefore, our results suggest that DIA might be a reliable method to assess proliferation index in patients affected with high risk PCa.

We observed a weak independent predictive role of Ki-67 positivity on BCR. These results are consistent with prior studies that have shown Ki-67 labeling index to be associated with BCR in prostate cancer patients treated with radical prostatectomy [23, 32–34], as recently highlighted in a large multicenter study [35]. The association with BCR was also observed using Ki-67 integrated in a three-marker composite index of proliferation [36], although it could be restricted to tumors with ERG- status [37]. One strength of the current study is that this association was observed for both a dichotomous (≤1.7 % or >1.7 %) as well as for a continuous measurement of Ki-67. The low cut-off point obtained could be related to TMA sampling or the sharp tissue segmentation we aimed at and the fact that we performed an exhaustive count of tumor cells rather than a «hot spot» approach, which might be factors preventing the overestimation of the proliferative index. We are confident that the data generated are valid because they were confirmed by two modalities of quantification (visual scoring and DIA).

In our cohort, Ki-67 was a much more potent predictor of DPCa than of BCR. When looking only at cohorts of patients undergoing radical prostatectomy, the association between Ki-67 and DPCa has been shown in some [16, 22] but not all [38] studies. Many studies considered only biochemical failure and did not assess DPCa, while others included patient populations with heterogeneous treatments or measured proliferative index in nodal metastases instead of primary tumors [39–41]. This is in contrast with the strong relationship between death and Ki-67 proliferative index observed in radiotherapy or watchful waiting cohorts (reviewed in Fisher 2013 [42]). Our own study confirmed that Ki-67 expression is positively associated with DPCa in a cohort of closely followed RP patients. Interestingly, when we considered the association between Ki-67 and death from other causes than prostate cancer, we found a negative association in multivariate analysis (HR 0.26, 95 % CI 0.13-0.77; p = 0.04). This observation suggests that Ki-67’s role in predicting death from prostate cancer might be very specific.

There are a growing number of reports of automated Ki-67 quantification data generated with software algorithms and of agreement with visual counts in several tissue and tumor types [43–46]. When different techniques of measurement of Ki-67 were compared in breast cancer, DIA was found to be even prognostically stronger than visual counting [31]. The use of DIA technique to quantify Ki-67 was also used successfully in PCa [24–26, 35, 47], but few studies have directly compared the two modalities. In one radiation therapy cohort measuring the proliferative index on prostate biopsies samples, there was a stronger correlation between clinical outcomes and visual scoring than with DIA [48]. This contrast with our results might reflect differences in sampling (biopsy vs TMAs), analysis (dichotomous vs continuous) and DIA methods. Our study also shows that the assessment of Ki-67 as a continuous variable is better than as a dichotomous variable because the HR estimates are better (the variance is smaller than in the dichotomous model), supporting the notion that capacity of acquiring continuous data is one advantage of the DIA technology. Finally, the algorithm used for our DIA measurements provided direct nuclei count with fractionation of staining in intensity classes. It allowed us to assess if a weighted H-score would perform better than raw percentage. Our results suggest that subtle intensity variations in Ki-67 staining, as detected with DIA and transposed into a H-score, adds no additional clinically useful information in prostate cancer.

The DIA system we used was successfully applied in morphologically complex tumors such as ovarian carcinomas [49], but resulted in high number of manual adjustments for tissue segmentation in our cohort of prostate carcinoma. This result reflects the subtleties in morphologic differences between malignant and benign glands in prostate cancer, as we aimed to perform sharp neoplastic gland segmentation, and the difficulty of DIA modalities to discriminate them without counter verification. It also underscores that an accurate assessment of tumor cells is mandatory when faced with a tumor with a low mean value of proliferative cells, since small variations may have a larger impact on data interpretation. Present estimations of the time needed for a pathologist to make a careful objective count is approximately 20 minutes, similar to the estimated time needed to perform DIA [31], especially if manual correction of tissue segmentation is required. It is reasonable to expect that with experience and availability of new softwares, quantitative DIA will be easier to apply and will lead to more constant and reproducible results, as the machine is not subject to fatigue nor to inter and intra observer variability. In addition, it remains that pre-analytical aspects inherent to tissue processing and immunohistochemistry techniques are also critical to optimize the performance of DIA [29].

There are potential limitations to our study that merit consideration. First, because of the relatively small number of patients, we could not perform a validation step. Therefore, the results obtained with our cohort of 225 patients will need to be validated in an independent cohort of patients. Second, the relatively high rate of manual adjustments of segmentation with the DIA technique certainly contributed to increase the precision of our findings but increased the technical burden of our approach. More methodological work is justified and needed to facilitate the application of our approach to the clinic. We also observed a low level of DPCa in our cohort. In order to compensate for this, an analysis taking the occurrence of positive margins and nodal involvement (high risk features) as a surrogate marker of dismal evolution was performed and reproduced our results (multivariate model; HR 3.49, 95 % CI 1.19-10.23; see Table 2). In addition, most of the patients in our cohort had a high stage (T3) tumor, and our results should be validated in a cohort with a more evenly distributed pathological stage to ensure that our findings are generalizable to lower stage prostate cancer patients. However, 31 % of such low-stage patients were included in our cohort and our analysis account for the stage effect, rendering confounding by stage unlikely. Finally, the use of TMAs rather than whole tissue sections might limit the transposition of our results to prostatectomy specimens. Tissue microarrays offer a tissue sampling independent of biomarker’s expression, thus reducing the opportunity for selection bias (e.g. of counting areas) and allows for a reduction in the number of immunohistochemistry runs, conferring more homogenous conditions for marker quantification. For Ki-67 measurements in PCa, specifically, it was suggested that as few as three TMA spots were sufficient to predict PSA failure [50]. However, since prostatectomy whole tissue sections are more convenient for direct clinical use, a validation of our results with such specimens should be done.

## Conclusions

In summary, our results show that the digital image analysis method we developed is comparable to visual scoring for quantifying Ki-67 expression in prostate cancer and predicting important clinical outcomes. Also, they show that the use of Ki-67 as a continuous variable is more reliable than using the traditional dichotomous approach. With improved standardization, DIA could become a clinically useful mean of measuring Ki-67, a proliferating marker associated with risk of BCR and DPCa in prostate cancer patients.

## Additional files

Additional file 1:
**Figure S1.** A. Representative prostate carcinoma TMA spot with Ki-67 immunostaining. B. The corresponding result of automated tissue segmentation and analysis of nuclei staining by digital image analysis is shown; Pink = tissue mask; circles: blue = no staining detected; yellow, orange and red = light, moderated and high levels of intensity recognized by the software, respectively. (TIFF 2931 kb) Additional file 2:
**Figure S2.** Log-linearity test for the covariates used in the Cox regression model for biochemical recurrence. The log-linearity of the four covariates, i.e. Ki-67 (A), PSA (B), Gleason score (C) and age (D) was assessed. The red line in each graph represents the perfect log-linearity while the black line shows the behavior of each variable. Only Ki-67 deviates from log-linearity. Additional file 3:
**Figure S3.** Transformation of Ki-67 to reach log-linearity in the Cox regression model for biochemical recurrence. As Ki-67 was shown not to be log-linear, a square root transformation was performed (Ki-67*). The red line in each graph represents the perfect log-linearity while the black line shows the behavior of each variable. As shown in panel A, Ki67 respected the log-linearity assumption of the Cox regression model for biochemical recurrence after a square root transformation. Additional file 4:
**Figure S4.** Log-linearity test for the covariates used in the Cox regression model for death by prostate cancer. The log-linearity of the four covariates, i.e. Ki-67 (A), PSA (B), Gleason score (C) and age (D) was assessed. The red line represents the perfect log-linearity while the black line shows the behavior of each variable. Additional file 5
**Table S1**: Verification of the proportionality assumptions for the covariates used in the Cox regression models. Verification of the proportionality assumptions for the covariates used in the Cox regression model for biochemical recurrence and death by prostate cancer. All covariates verified the assumption.

## Abbreviations

DIA: Digital image analysis
BCR: Biochemical recurrence
PCa: Prostate cancer
DPCa: Death by prostate cancer
VS: Visual scoring
HR: Hazard ratio
RP: Radical prostatectomy
TMA: Tissue microarray
SD: Standard deviation
IQR: Interquartile range
