# Supplementary figures and images for: Comparison of digital image analysis and visual scoring of KI-67 in prostate cancer prognosis after prostatectomy
Source: Diagn Pathol. 2015 Jun 13;10:67. doi: 10.1186/s13000-015-0294-0 (PMC4465166; doi:10.1186/s13000-015-0294-0)

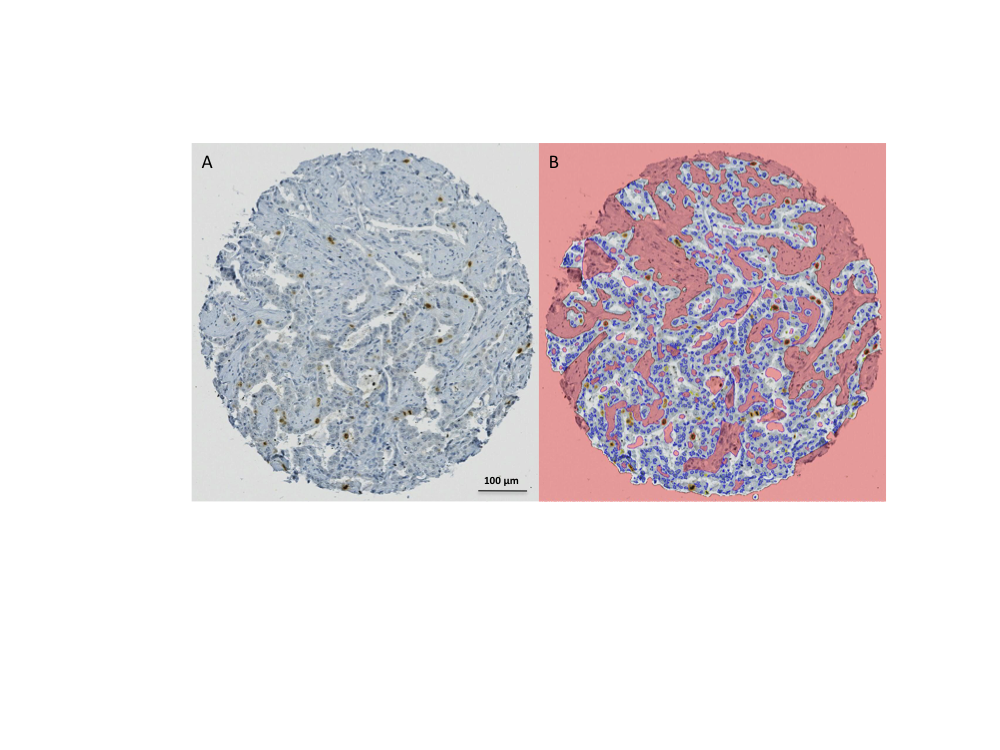

Supplement: Additional file 1: — Figure S1. A. Representative prostate carcinoma TMA spot with Ki-67 immunostaining. B. The corresponding result of automated tissue segmentation and analysis of nuclei staining by digital image analysis is shown; Pink = tissue mask; circles: blue = no staining detected; yellow, orange and red = light, moderated and high levels of intensity recognized by the software, respectively. (TIFF 2931 kb) [file 13000_2015_294_MOESM1_ESM.tiff]

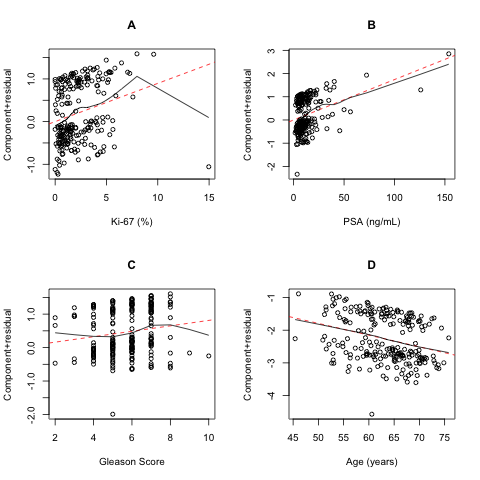

Supplement: Additional file 2: — Figure S2. Log-linearity test for the covariates used in the Cox regression model for biochemical recurrence. The log-linearity of the four covariates, i.e. Ki-67 (A), PSA (B), Gleason score (C) and age (D) was assessed. The red line in each graph represents the perfect log-linearity while the black line shows the behavior of each variable. Only Ki-67 deviates from log-linearity. [file 13000_2015_294_MOESM2_ESM.tiff]

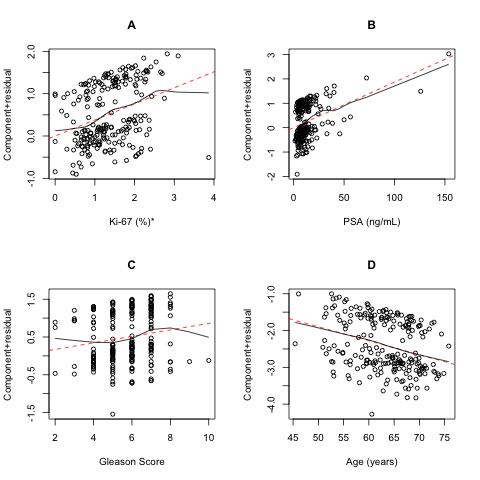

Supplement: Additional file 3: — Figure S3. Transformation of Ki-67 to reach log-linearity in the Cox regression model for biochemical recurrence. As Ki-67 was shown not to be log-linear, a square root transformation was performed (Ki-67*). The red line in each graph represents the perfect log-linearity while the black line shows the behavior of each variable. As shown in panel A, Ki67 respected the log-linearity assumption of the Cox regression model for biochemical recurrence after a square root transformation. [file 13000_2015_294_MOESM3_ESM.tiff]

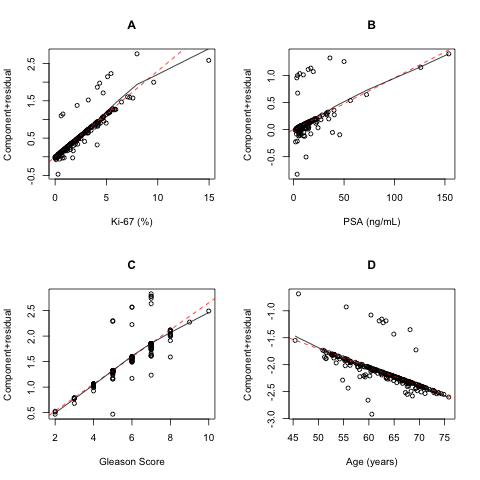

Supplement: Additional file 4: — Figure S4. Log-linearity test for the covariates used in the Cox regression model for death by prostate cancer. The log-linearity of the four covariates, i.e. Ki-67 (A), PSA (B), Gleason score (C) and age (D) was assessed. The red line represents the perfect log-linearity while the black line shows the behavior of each variable. [file 13000_2015_294_MOESM4_ESM.tiff]
