# Supplementary material for: Comparison of digital image analysis and visual scoring of KI-67 in prostate cancer prognosis after prostatectomy
Source: Diagn Pathol. 2015 Jun 13;10:67. doi: 10.1186/s13000-015-0294-0 (PMC4465166; doi:10.1186/s13000-015-0294-0)
Supplement: Additional file 5 — Table S1: Verification of the proportionality assumptions for the covariates used in the Cox regression models. Verification of the proportionality assumptions for the covariates used in the Cox regression model for biochemical recurrence and death by prostate cancer. All covariates verified the assumption. [file 13000_2015_294_MOESM5_ESM.docx]

| **Supplementary Table 1:** Verification of the proportionality assumptions for the covariates used in the Cox regression models | | |
| --- | --- | --- |
| ***Outcome: Biochemical recurrence*** | | |
| ***Covariate*** | ***Χ^2^*** | ***p-value*** |
| Ki-67* | 0.324 | 0.569 |
| PSA (μg/L) | 1.099 | 0.294 |
| Gleason Score | 0.001 | 0.967 |
| pT3N+M+ | 1.540 | 0.215 |
| Age (years) | 0.056 | 0.812 |
| Global | 2.755 | 0.738 |
| ***Outcome: Death from prostate cancer*** | | |
| ***Covariate*** | ***Χ^2^*** | ***p-value*** |
| Ki-67 | 1.966 | 0.161 |
| PSA (μg/L) | 1.264 | 0.261 |
| Gleason Score | 0.468 | 0.494 |
| pT3N+M+ | 1.125 | 0.724 |
| Age (years) | 0.408 | 0.523 |
| Global | 3.099 | 0.685 |
| Ki-67*: The square root of Ki-67;  PSA: Prostate-specific antigen; N+: nodal involvement; M+: positive margins | | |
